# Colonization by native species enhances the carbon storage capacity of exotic mangrove monocultures

**DOI:** 10.1186/s13021-020-00165-0

**Published:** 2020-12-14

**Authors:** Ziying He, Huaye Sun, Yisheng Peng, Zhan Hu, Yingjie Cao, Shing Yip Lee

**Affiliations:** 1 School of Marine Science, Southern Marine Science and Engineering Guangdong Laboratory (Zhuhai), Sun Yat-sen University, Guangzhou, 510275 China; 2 School of Environmental Science and Engineering, Sun Yat-Sen University, Guangzhou, 510275 China; 3 Guangdong Provincial Key Laboratory of Environmental Pollution Control and Remediation Technology, Sun Yat-Sen University, Guangzhou, 510275 China; 4 Southern Marine Science and Engineering Guangdong Laboratory, Sun Yat-Sen University, Zhuhai, 519000 China; 5 Simon F. S. Li Marine Science Laboratory, School of Life Sciences, The Chinese University of Hong Kong, Hong Kong SAR, China

**Keywords:** Mangrove plantation, Carbon storage, Mixed forest, *Kandelia obovata*, *Sonneratia apetala*

## Abstract

**Background:**

The fast-growing introduced mangrove *Sonneratia apetala* is widely used for mangrove afforestation and reforestation in China. Some studies suggested that this exotic species outperforms native species in terms of carbon sequestration potential. This study tested the hypothesis that multi-species mangrove plantations might have higher carbon sequestration potential than *S*. *apetala* monocultures.

**Results:**

Our field measurements at Hanjiang River Estuary (Guangdong province, China) showed that the carbon stock (46.0 ± 3.0 Mg/ha) in *S*. *apetala* plantations where the native *Kandelia obovata* formed an understory shrub layer was slightly higher than that in *S*. *apetala* monocultures (36.6 ± 1.3 Mg/ha). Moreover, the carbon stock in monospecific *K. obovata* stands (106.6 ± 1.4 Mg/ha) was much larger than that of *S*. *apetala* monocultures.

**Conclusions:**

Our results show that *K. obovata* monocultures may have a higher carbon accumulation rate than *S*. *apetala* monocultures. Planting *K. obovata* seedlings in existing *S*. *apetala* plantations may enhance the carbon sink associated with these plantations.

## Background

Although covering only 0.1% of Earth’s continental surface, mangrove forests are amongst the most carbon-rich ecosystems in the world [1, 2]. Mangrove forests differ from terrestrial counterparts in their capacity to store  > 90% of their carbon in the substrate (5–10.4 Pg globally) over millennial timescales [3–5]. Complex root structures, high sedimentation rates, periodically inundated conditions and muddy anaerobic soils are responsible for exponentially higher carbon burial rates and millesimal lower soil carbon turnover rates in mangroves compared to those of terrestrial forests [6–8]. The high carbon sequestration and storage rates are among the mangrove ecosystem services focused including coastal protection, sediment retention, and nurseries for marine fishery species [1, 9–11]. Despite their ecological importance, mangroves are encountering a multitude of anthropogenic threats such as coastal development or pollution, leading to their widespread degradation and decline [12–14]. Mangroves have been significantly deforested during the last several decades in China. The total area of mangrove in China was estimated  ~ 20,303 ha in 2015, representing less than one-third of the 1950s [15, 16]. Hence, large investment and great efforts have been paid in mangrove afforestation/reforestation to mitigate the adverse impacts of mangrove loss on biodiversity, ecosystem stability, and carbon sequestration since 1980s in China [16].

The largest carbon pools are associated with living tree biomass and soil organic matter in forest ecosystems [17–19]. Thus, carbon stock in these compartments determines the carbon accumulation capacity of mangrove ecosystem. In contrast to terrestrial forests, mangroves allocate a high proportion of their biomass to belowground components, representing more than half of the total standing biomass [20, 21]. Carbon stored in belowground biomass varied among mangrove species and more than 50% of mangroves soil carbon were plant-derived [20, 22–25]. The amount of soil organic carbon of mangrove forests dominated by different species varies greatly, from less than 0.5% to 40%, with a global mean of 2.2% [26].

Strategies for afforestation have primarily relied on monocultures with low biodiversity, ecological value, and potentially lower capacity for carbon sequestration [27, 28]. Most countries including China have promoted monocultures in many mangrove reforestation/afforestation programs by simplistic seedling plantings with dubious long-term survival [29]. Even when successfully established, planting programs often lack adequate cost–benefit and ecosystem services evaluation at the ecosystem level [30]. *Sonneratia apetala* was native to Bangladesh that introduced to China in 1985 and has been planted extensively in mangrove afforestation programs [31, 32]. In China, the extent of *S. apetala* forests is estimated at  > 50% (3800 ha) of the total mangrove plantations area [24, 33, 34], while *Kandelia obovata,* a native mangrove species with the widest natural distribution and dominant along the southeast Chinese coastline, has also been widely planted for mangrove afforestation. Ren et al. [33] suggested that the fast-growing *S. apetala* had great potential on carbon sequestration than most of the native species, and should be preferred in afforestation [18, 33]. However, the capacity for carbon storage in mangrove ecosystems is species-dependent. Multi-specific plantations have more capacity for carbon sequestration in both biomass and soil, compared to monocultures [27, 35], and are recommended for the implementation of mangrove afforestation programmes. Peng et al. [36] also suggested that native mangrove species can be planted into existing *S. apetala* plantations to establish mixed stands for enhancing functional diversity and provide more ‘complete’ services than monospecific forests. However, potential differences in the capacity for carbon sequestration between monospecific and multi-specific mangrove plantations are poorly known currently. In the present study, we compared the carbon storage capacity of monocultures of *S*. *apetala* (as SA below) and *K*. *obovata* (as KO below) as well as mixed stands of the two species (as KS below) in Hanjiang River Estuary, southern China. The physiological traits, aboveground biomass, belowground biomass and total carbon storage of the mangrove forests were compared to test the hypotheses that: (1) carbon storage is higher in monospecific *S. apetala* plantations than in monospecific *K*. *obovata* plantations due to their different growth traits; and (2) the mixed stands stored more carbon in both biomass and soil than the monocultures.

## Methods

### Study sites

**Fig. 1:**
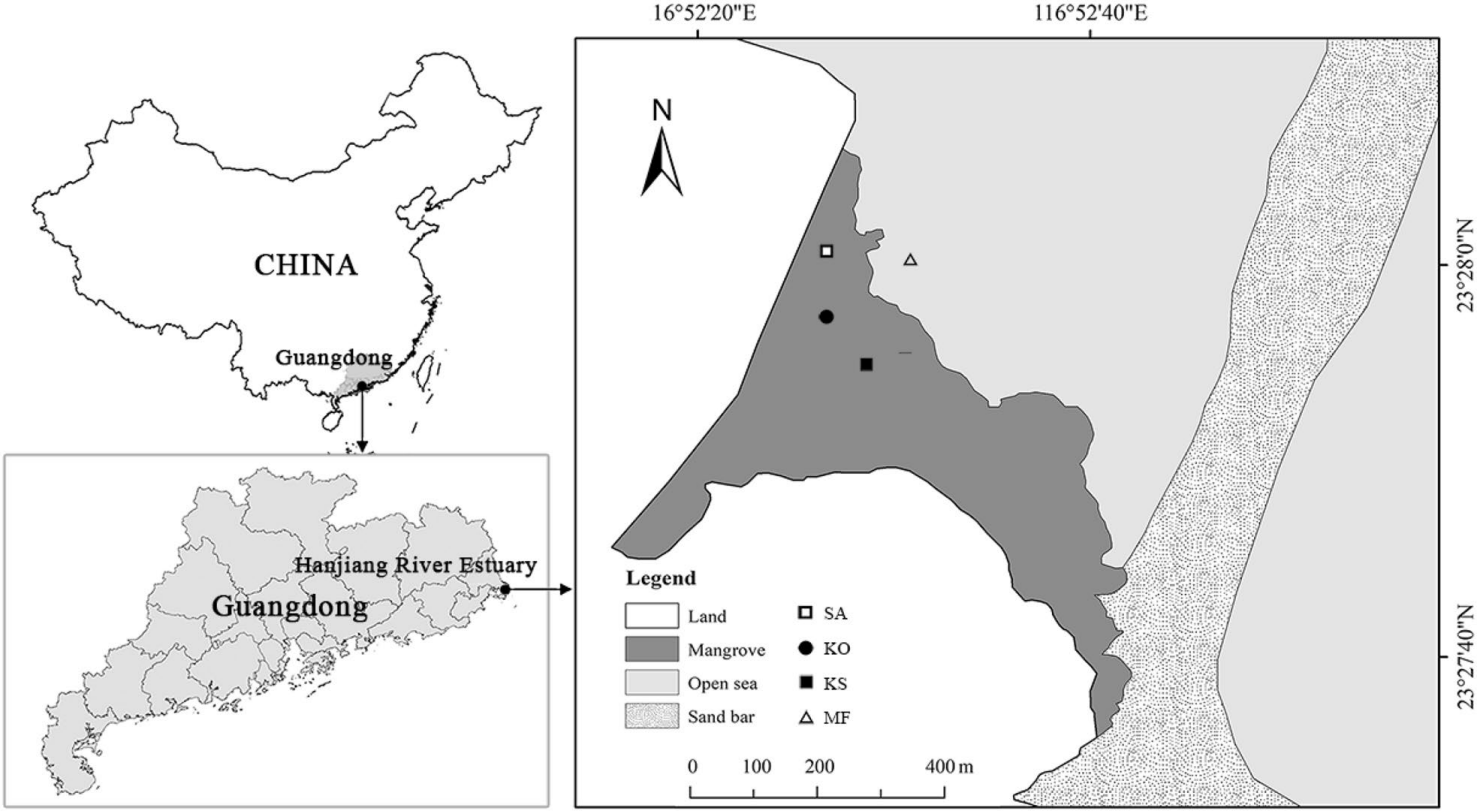
The location of sampling sites at Hanjiang River Estuary, Guangdong Province, southern China: *S. apetala* monoculture (SA), *K. obovata* monoculture (KO), mixed *K. obovata* and *S. apetala* (KS), and mudflat (MF)

This study was carried out at Hanjiang River Estuary (23°45′ N, 116°43′ E), Guangdong province, China (Fig. 1). The climate in Hanjiang River Estuary is principally influenced by monsoon, with annual precipitation of 1300-1800 mm, and a mean temperature of 21.3 °C. Semi-diurnal tides with a mean range of 1.35 m occur in the study area. In 2005, monospecific stands respectively of *S. apetala* and *K. obovata* were planted on unvegetated mudflats (as MF below) with an initial density of 2500 seedlings per hectare. After 12 years of development, *K. obovata* (KO) has gradually colonized into *S. apetala* (SA) forests naturally as an understory shrub layer and forming mixed stands with both *S. apetala* and *K. obovata* (KS).

### Field sampling and laboratory analysis

In November 2016, we sampled in KO, SA, and the mixed KS forests with nine plots (10 m × 10 m) in total for aboveground biomass. Tree height, tree density and the stem diameter at breast height (DBH, at 1.3 m) were measured in these plots except for *K. obovata* individuals in plots of KS, due to the infeasibility of measuring DBH because of its shrubby growth form. Tree basal area (BA) was derived from DBH measurements [37]. Species-specific allometric equations were using for calculated the aboveground biomass of KO, SA, and *S. apetala* in KS [32, 38]. For estimating *K. obovata* aboveground biomass in KS, branches and leaves were harvested from three standard trees that randomly selected in each plot. The stem biomass was determined from the product of stem volume and wood density of each sampled tree. Stem volume was measured following the method of Kamal et al. [39] using an Xbox360 Kinect for Windows (Microsoft Inc., USA). The specific stem wood density was determined using stem sections wood samples taken at the base of each sampling tree [40]. The volume of each sample was determined by the water volume displaced when submerged. The specific wood density was calculated as the ratio of oven dried weight (65 °C, 72 h) to the volume. The aboveground biomass of *K. obovata* species in KS was calculated by summing the biomass of harvested components and stems.

To determine ground layer biomass, seedlings and litter from five 1 m × 1 m plots at the three mangrove plantations were collected, then oven dried and weighed. For belowground biomass, root coring was conducted at the three mangrove forests. Three standard trees were randomly selected for root coring within each plot. One soil core was taken with a PVC tube of 1 m in depth at the position of mid-canopy of each tree. In order to minimize the soil compression effect, the PVC tube with a larger diameter (11 cm) was used. The cores were average divided into five vertical sections. Each root core sample was put on a 0.25 mm mesh sieve and washed with tap water. Live and dead roots were then separated using 11% and 6% colloidal silica solutions (Ludox^®^ TM, Sigma-Aldrich Inc., USA) [41]. All roots samples were then dried at 65 °C to a constant weight and reweighed. All calculations were based on the dry weights [20, 37].

For determination of soil organic carbon content, soil samples were collected by coring at the mangrove forests and the adjacent mudflat. Five cores from each sampling site at the KO, SA plantations and MF (as a control to assess the effect of mangrove afforestation on carbon sequestration), and eight cores at KS were randomly collected. The cores were sectioned to five layers (0–20, 20–40, 40–60, 60–80 and 80–100 cm). Soil samples were then air-dried for the following laboratory analyses.

Dry biomass samples (leaves, branches, stems, and roots) and soil samples were grounded. Soil samples were processed by the modified Walkley–Black method for determining soil organic carbon content [24, 42, 43]. Biomass samples were analyzed for organic carbon contents using the loss-on-ignition method [24, 44]. The total organic carbon contents of the sample trees were calculated by multiplying the biomass of branches, leaves, flowers, fruits stems and roots by their respective organic carbon contents. The sum of branch, leaf and stem carbon is taken as the aboveground biomass organic carbon. The ground layer biomass organic carbon was estimated by multiplying the dry mass of collected seedlings and litter from each quadrat and their respective organic carbon concentrations. The belowground organic carbon is represented by the root organic carbon storage. The soil organic carbon content in each quadrat was used for estimating soil organic carbon stock of specific layers. The organic carbon stock of soil layers were then summed to estimate the soil organic carbon storage to 1 m for each sampling site. The total organic carbon storage at each plot was estimated as the sum of the vegetation organic carbon storage, ground layer organic carbon storage, and soil organic carbon storage.

### Statistical analysis

All statistical analyses were performed by SPSS software (version 23.0, SPSS Inc., USA). Significance was determined at α = 0.05. One-way analysis of variance (ANOVA) was used to test for differences in forest structure, plantation biomass and organic carbon storage across forest types. Differences in soil parameters at different depths among the four study sites were also analyzed by one-way ANOVA. Two-way ANOVA was applied to test the difference in soil parameters, root biomass and organic carbon storage in roots and litter among habitats and soil depths. The relationship between organic carbon storage in litter, roots, and soil was explored by linear regression analysis.

## Results

### Forest structure and biomass

Stem density significantly differed among the three mangrove forests (*p *< 0.001). The KO plantation had the highest stand density, while its average stem diameter and height were significantly lower than those of the other two forests (*p *< 0.001; Table 1). Natural colonization of *K*. *obovata* increased the stand density of KS markedly from 1367 to 2600 stem ha^−1^ over the history of forest. Tree height and basal area of *S. apetala* showed no significant differences between the monoculture and mixed forest, while such parameters of *K*. *obovata* in the KO plantation were significantly lower than those of *S. apetala* in both SA and KS.

**Table 1:** Forest structure and biomass of different mangrove forest types in Hanjiang River Estuary

Parameters	Forest type		
	SA	KO	KS
Density (stems ha^−1^)	1367 ± 145^a#^	9533 ± 63^c#^	2600 ± 557^b^
DBH (cm)	14.5 ± 0.9^b#^	8.8 ± 0.3^a#^	16.3 ± 0.9^b^*
Tree height (m)	8.5 ± 0.3^b#^	4.8 ± 0.07^a#^	8.8 ± 0.3^b^*
Basal area (m^2^ ha^−1^)	26.0 ± 3.0^a#^	62.7 ± 4.5^b#^	27.8 ± 2.8^a^*
Aboveground biomass (Mg ha^−1^)	67.1 ± 7.7^a^	195.8 ± 23.7^b^	71.9 ± 11.7^a^
Ground layer biomass (Mg ha^−1^)	2.9 ± 0.6^b^	1.5 ± 0.3^a^	2.8 ± 0.5^b^
Root biomass (Mg ha^−1^)	3.7 ± 0.2^a^	34.0 ± 3.6^c^	11.9 ± 1.3^b^
Total vegetation biomass per unit stem (kg)	53.9 ± 3.9^a^	24.3 ± 1.5^c^	33.3 ± 3.2^b^

Data are mean ± SE, n = 3 to 5. Different letters indicate significant differences among the three plantations (*p *< 0.05)

Different superscripts in the same column indicate significant difference across forest types (*p *< 0.05)

* Data only refers to *S. apetala* species in the mixed KS forest

^#^Data source: He et al. [24]

**Fig. 2:**
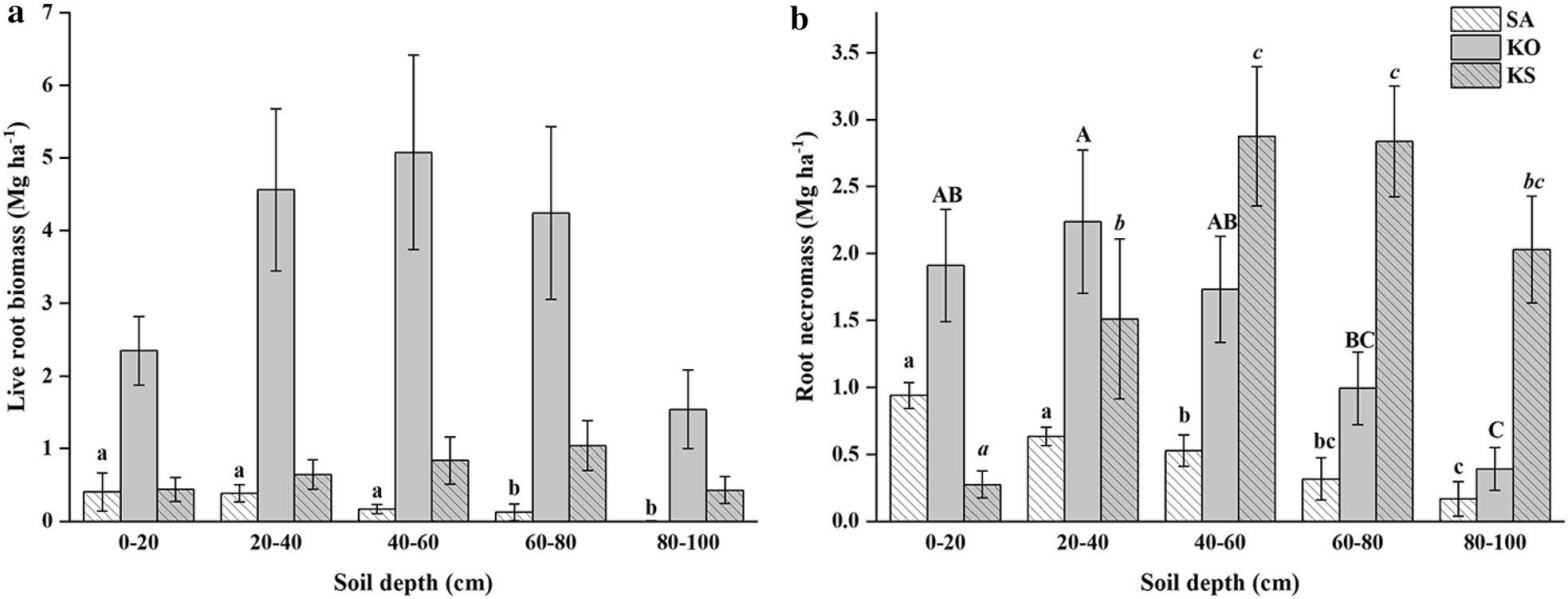
Vertical distributional patterns of live root biomass (**a**) and root necromass (**b**) (mean ± 1SE) in *S. apetala* (SA) and *K. obovata* (KO) monocultures and mixed forest (KS) at Hanjiang River Estuary, south China. Different letters of the same font indicate significant differences among different soil depths within the forest (*p *< 0.05)

The highest mean total vegetation biomass occurred in KO (231.3 ± 14.7 Mg ha^−1^), followed by KS and SA at 86.6 ± 8.2 and 73.7 ± 5.4 Mg ha^−1^, respectively. However, the total vegetation biomass per unit stem of the two forests with *S. apetala* present were both significantly higher than that of *K*. *obovata* monoculture (*p *< 0.001; Table 1). The aboveground and ground layer biomass significantly differed across forest types (*p *< 0.05; Table 1), while this profile also applied to total root biomass, which ranged from 3.7 ± 0.2 to 34.0 ± 3.6 Mg ha^−1^ (*p *< 0.001; Table 1). In SA, live root biomass was negatively correlated with soil depth (*p *= 0.002; Fig. 2a). Similarly, root necromass in SA significantly decreased with soil depth (*p *= 0.003; Fig. 2b). No significant difference was detected in live root biomass among soil depths in KO, while the root necromass of this species showed a similar distributional pattern in depth as that of SA (*p *= 0.01; Fig. 2b). In KS, live root biomass did not differ among soil depths, while root necromass increased with soil depth (*p *< 0.001; Fig. 2b). The total live root biomass was 1.1 ± 0.2, 17.8 ± 3.9 and 3.4 ± 0.6 Mg ha^−1^ in SA, KO and KS, respectively, accounting for 29.7%, 52.3% and 28.7% of total root biomass. There were significant differences between both plantation types and soil depths (*p *< 0.001). Forest types and soil depth also interacted significantly in terms of live root biomass in the three mangrove forests (*p *= 0.015; Table 2). The overall root necromass of KO and KS were 7.3 ± 1.4 and 9.5 ± 1.4 Mg ha^−1^, respectively, which were significantly higher than that of SA. There were significant differences of root necromass between both forest types and soil depths (*p *= 0.004 and 0.008, respectively; Table 3), also with significant interaction effects (*p *< 0.001; Table 2).

**Table 2:** *F* values of two-way ANOVA testing the differences in live roots biomass, roots necromass and soil variables among different mangrove forest types/sites and soil depth in Hanjiang River Estuary, south China

Dependent variables	Sources of variance		
	Forest types/Sites	Soil depth	Forest types/Sites × Soil depth
Live roots biomass (Mg ha^−1^)	33.454**	5.058**	7.429*
Root necromass (Mg ha^−1^)	5.127*	4.162*	0.738**
Organic carbon storage in live roots (MgC ha^−1^)	34.316*	5.812**	2.804**
Organic carbon storage in dead roots (MgC ha^−1^)	15.063**	2.227	5.162**
Organic carbon storage in soil (MgC ha^−1^)	2.659*	34.167**	6.629**

* *p *< 0.05 (n = 75 to 85); ** *p *< 0.001 (n = 75 to 85)

**Table 3:** Organic carbon storage in different component of different habitats in Hanjiang River Estuary

Parameters	Habitats			
	SA	KO	KS	MF
AGOC (MgC ha^−1^)	26.1 ± 0.8^b^	73.6 ± 1.3^a^	27.3 ± 0.7^b^	NA
BGOC (MgC ha^−1^)	1.2 ± 0.1^c^	11.5 ± 1.0^a^	5.2 ± 0.5^b^	NA
AGOC:BGOC	21.9^a^	6.4^b^	5.3^c^	NA
GLOC (MgC ha^−1^)	1.5 ± 0.3^a^	0.7 ± 0.1^b^	1.6 ± 0.2^a^	NA
SOC (MgC ha^−1^)	7.8 ± 0.5^c#^	15.8 ± 0.8^a#^	11.9 ± 2.4^b^	4.7 ± 0.9^d#^
TOC (MgC ha^−1^)	36.6 ± 1.3^c^	101.6 ± 1.4^a^	46.0 ± 3.0^b^	4.7 ± 0.9^d^
TOC per unit stem (kgOC)	26.8 ± 8.9^a^	10.7 ± 2.2^c^	17.7 ± 5.4^b^	NA

Data are mean ± SE, n = 3 to 5

Superscripts in the same column indicate significant difference across forest types (*p *< 0.05). AGOC, BGOC, GLOC, SOC and TOC refer to aboveground, belowground, ground layer, soil, and total organic carbon storage, respectively

^#^Data source: He et al. [24]

### Organic carbon stocks and accumulation

**Fig. 3:**
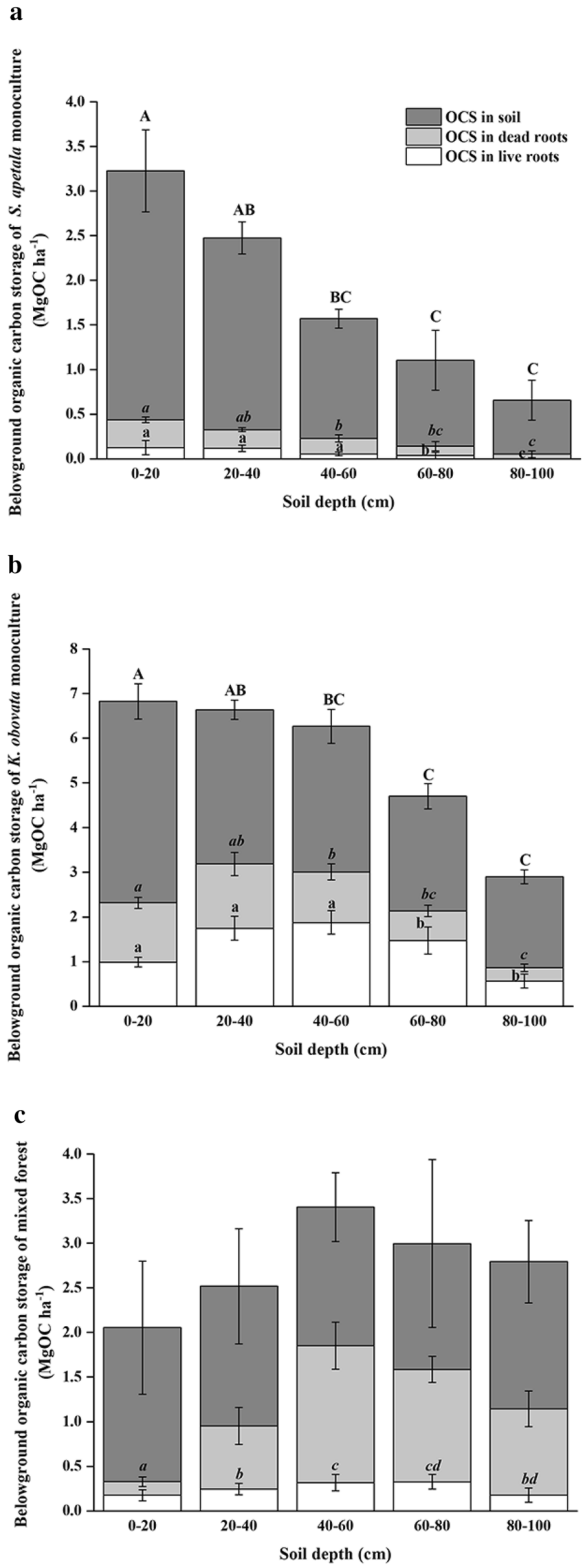
Belowground organic carbon storage of *S*. *apetala* (**a**), *K. obovata* (**b**) monocultures and mixed forest (**c**) (mean ± 1SE) at Hanjiang River Estuary, south China. Different letters in of the same front indicated significant differences among the different soil depths (*p *< 0.05). OCS refers to organic carbon storage

The total vegetation organic carbon storage of these three mangrove forests was significantly different, with KO (85.8 ± 1.4 MgC ha^−1^) being 2.5 and 3.0 times higher than those of KS and SA, respectively. The organic carbon storage in aboveground biomass of KO was significantly higher than those of SA and KS (*p *< 0.001; Table 3). Ground layer organic carbon storage accounted for 0.7%–4.1% of the total organic carbon storage, and the ground layer organic carbon storage peaked at KS (*p *= 0.019; Table 3). For belowground biomass, the maximum value was 15.8 ± 0.8 MgC ha ^−1^ at KO, which was significantly higher than those of the other two forests (*p *< 0.001; Table 3). Ratios of organic carbon storage in aboveground to belowground biomass may provide an indication of the proportion of C allocated to above- and belowground components. These ratios were 21.9 in SA, 6.4 in KO and 5.3 in KS, and were significantly different. Organic carbon storage in live roots varied with depth and the mean values were 0.3 ± 0.06, 6.7 ± 0.8 and 1.3 ± 0.2 MgC ha^−1^ in SA, KO and KS, respectively (Fig. 3). Significant differences in live root organic carbon storage among the three forest types and soil depths were detected (*p *< 0.001; Table 2). In SA and KO, the organic carbon storage in dead roots significantly decreased with soil depth (*p *= 0.002 or 0.009; Fig. 3a, b). In contrast, organic carbon storage in dead roots in KS increased with soil depth (*p *< 0.001; Fig. 3c). Organic carbon storage in live and dead roots of KO was significantly higher than those of SA and KS (*p*<0.001; Table 2). Plantation type and soil depth have significant interactive effects on organic carbon storage in live and dead roots of the three mangrove plantations (*p *< 0.01; Table 2).

The mangrove forests (0.96–3.3%) showed significantly higher soil organic carbon concentration than the mudflat (0.55%) 12 years after the mangrove planted and subsequent forest growth. The soil organic carbon (SOC) concentration (0–100 cm) of the mangrove forests varied between 0.96 ± 0.3% (He et al. [24] at SA and 3.3 ± 0.4% (He et al. [24] at KO, with the SOC concentration of both KO and KS (2.1 ± 0.4%) being significantly higher than that of SA (*p *< 0.001). The SOC concentration significantly decreased with soil depth for KO and SA but no significant trend was evident for KS and MF. Soil bulk density (SBD) showed the opposite trend to SOC concentration. The adjacent unvegetated mudflat had the highest SBD value for the entire 1 m soil column (0.94 ± 0.08 g cm^−3^; He et al. [24]) among the four study sites, which was close to that of SA (0.89 ± 0.04 g cm^−3^; He et al. [24]). The mean SBD (0–100 cm) of KO (0.45 ± 0.03 g cm^−3^; He et al. [24]) and KS (0.64 ± 0.05 g cm^−3^) were significantly lower than those of MF and SA (*p *< 0.001), indicating much finer sediment was found in these forests. The mean organic carbon storage in soil of KO was 15.8 ± 0.8 MgC ha^−1^, 2.01, 1.33 and 3.35 times higher than those of SA, KS and MF, respectively. Soil organic carbon storage was also significantly affected by site and soil depth, with a significant interaction effect (*p *< 0.01; Tables 2 and 3 and Fig. 3).

The total organic carbon storage of different habitats was estimated by summing the vegetation organic carbon storage, ground layer organic carbon storage, and soil organic carbon storage. In general, total organic carbon storage was highest in KO amongst all habitats, then decreasing significantly in the order KS, SA and MF (*p *< 0.001; Table 3). However, this trend was reversed in the individual tree level (Table 3).

### Organic carbon allocation patterns in different forests

**Fig. 4:**
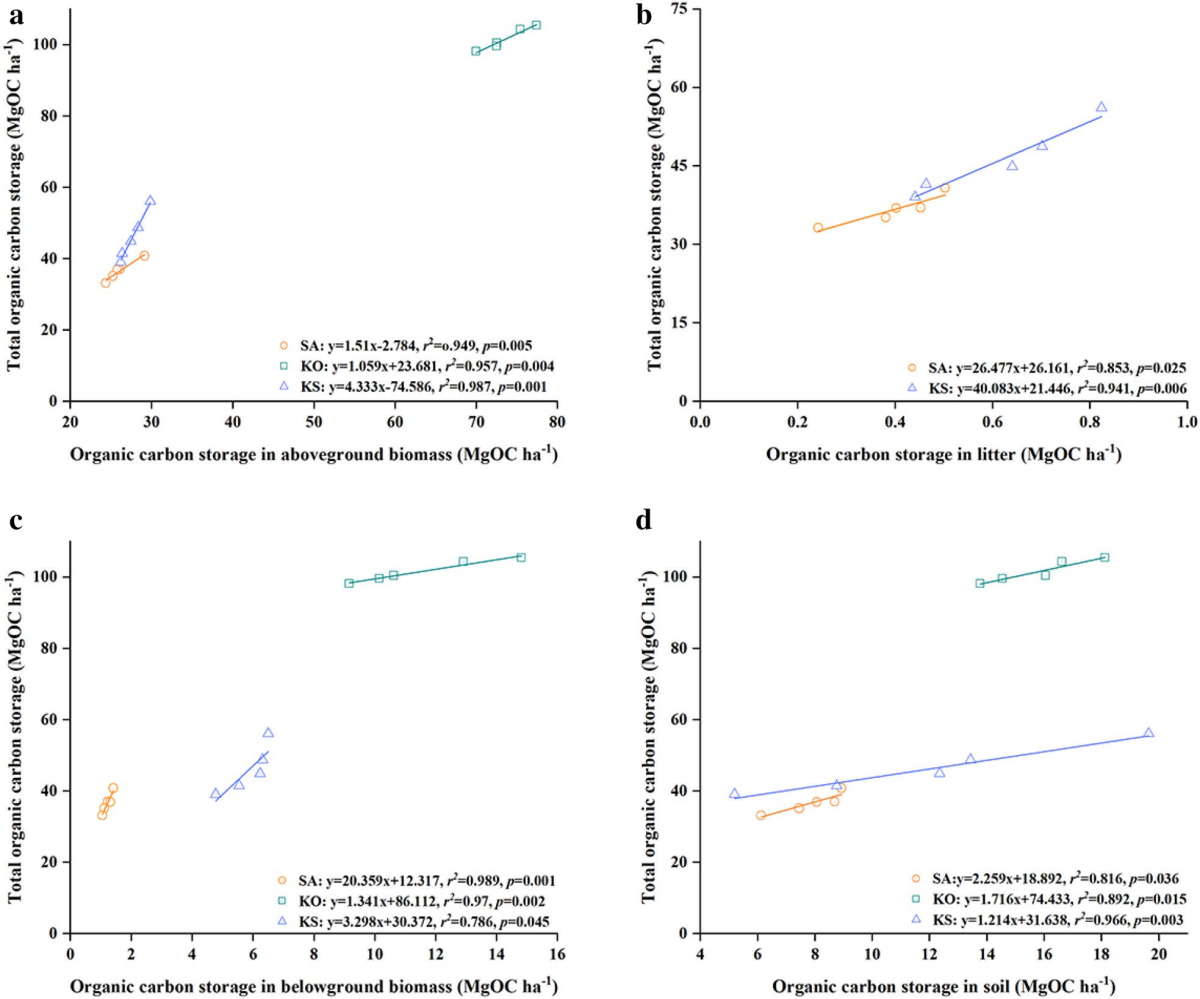
Regression analysis between total organic carbon storage and organic carbon storage in different components in the three mangrove forests in Hanjiang River Estuary, south China

Total organic carbon storage in all mangrove forests was positively correlated with organic carbon storage in aboveground biomass, belowground biomass, and soil (*p *< 0.05; Fig. 4). Moreover, in SA and KS, a significant positive correlation was found between organic carbon storage in litter and total organic carbon storage, while no such relationship was detected in KO, suggesting that roots contributed mostly to forest total organic carbon stock in KO (*p *< 0.01; Fig. 4).

**Fig. 5:**
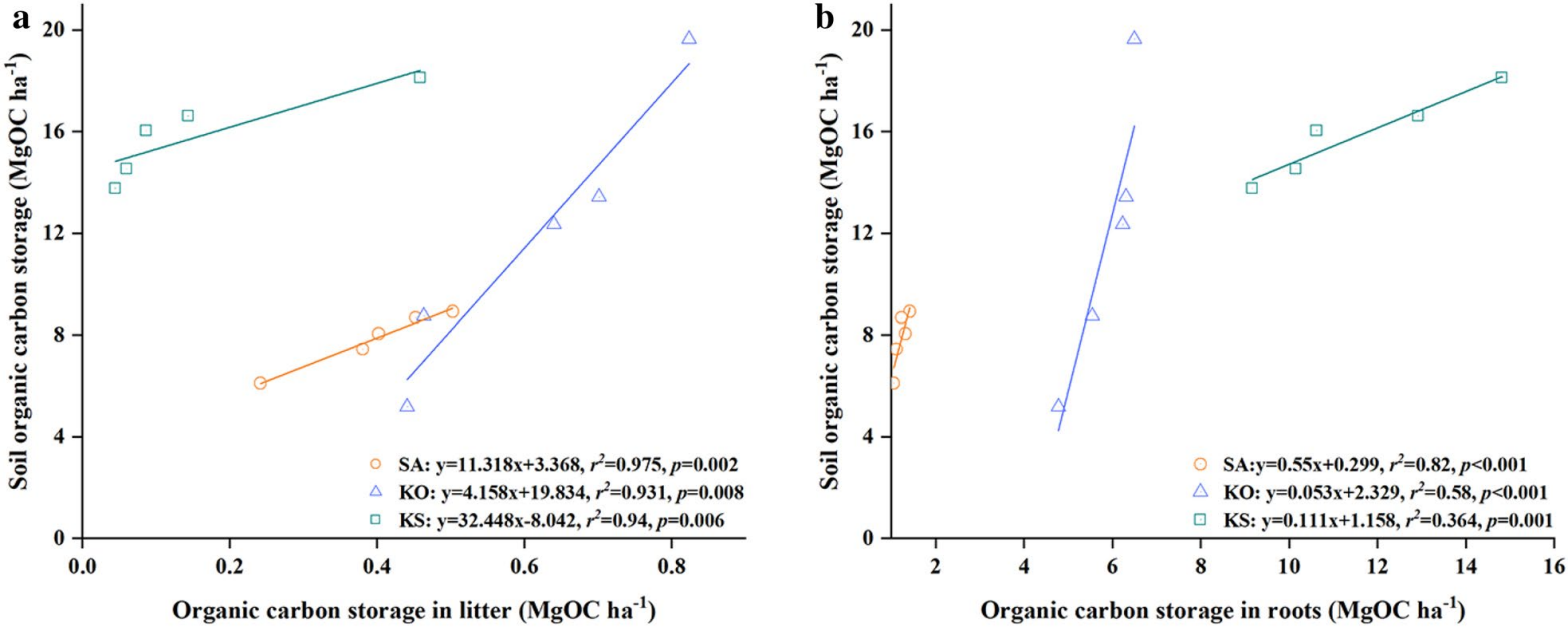
Regression analyses between soil organic carbon storage and organic carbon storage in litter and roots in the three mangrove forests in Hanjiang River Estuary, south China

Due to their vital contribution to soil organic carbon storage, the correlation among litter, root and soil organic carbon had also explored. The highest value of organic carbon storage in litter was found in KS (0.6 ± 0.07 MgC ha^−1^) and the lowest in KO (0.15 ± 0.08 MgC ha^−1^). Soil organic carbon density in all forests was significantly positively correlated to organic carbon storage in litter, and the slope of the regression line of SA and KS were significantly steeper than that of KO (*p *< 0.01; Fig. 5). Similarly, in all forests, a significant positive correlation was detected between organic carbon storage in soil and roots. The slopes of the regression lines are all significantly different, suggesting that the contribution of root organic carbon to soil organic carbon is dependent on stand composition (*p *< 0.001; Fig. 5).

## Discussion

### Organic carbon accumulation varies with different mangrove species

In most terrestrial forests, the largest carbon pools are associated with aboveground biomass and soil organic matter, with lesser contributions from roots and ground layer detritus [17]. Fast-growing species accumulate more carbon than slow-growing ones [45], which is consistent with our result that the individual biomass organic carbon stock in *S*. *apetala* monoculture was significantly higher than that of *K. obovata* monoculture. However, due to the characteristics of fast growth and intolerance to canopy shade of *S. apetala*, intense self-thinning occurs to result in lower tree density in the SA monoculture [32]. This development pattern explains the significant lower total biomass organic carbon stock of SA compared to KO at the forest level, despite the larger individual tree size in the former species.

The mixed forest of *K. obovata* and *S*. *apetala* (KS) has intermediate organic carbon biomass levels compared with the KO and SA monocultures. Colonization by *K. obovata* in the understory enhances the organic carbon storage in the *S*. *apetala* dominated plantation. In KS, growth of *S. apetala* individuals as reflected by tree height and basal stem diameter was not significantly different with those in SA. When the native *K. obovata* recruited naturally into the *S. apetala* plantation, the faster-growing *S. apetala* occupied the higher spatial niche, whereas *K. obovata* formed a lower shrub layer, indicating the native *K. obovata* is more shade-tolerant than the exotic and fast-growing *S. apetala* [46]. Despite the spread and colonisation of *K. obovata* significantly increased the stem density of KS, organic carbon storage in aboveground and ground layer biomass of KS was not significantly different from the SA monoculture. The difference in biomass organic carbon storage between KS and SA can be attributed to different root biomass between the two forests. In the monocultures, both root biomass and root organic carbon stock of *S*. *apetala* individuals were significantly higher than those of *K. obovata*. However, when the effect of density is considered, such pattern is reversed, i.e. SA supported significantly lower root biomass and organic carbon stock than KO did. Further, live root biomass and root necromass in SA decreased with soil depth. This might reflect the faster decomposition rates of *S. apetala* roots compared with those of *K. obovata* [20, 47].

*Sonneratia apetala* had significantly higher live root biomass, root necromass and root organic carbon storage in the mix forest (KS) than in the SA monoculture. The colonisation of *K. obovata* is associated with a different profile in root growth in *S. apetala*, which allowed the complementary vertical niche differentiation in belowground space utilisation in this mixed-species zone. The taller *S*. *apetala* was characterized with deeper root system in soil, while the shorter *K*. *obovata* occupied the understory of forest and developed shallower roots in soil. Positive interactions in mixed species forests may increase productivity beyond that of monospecific stands [48]. Our findings suggest different interaction outcomes, i.e. positive and negative effects on individual biomass, respectively, for *S*. *apetala* and *K. obovata*, in the mixed forest [48]. A 25-month experiment showed that a mixed stand of *S*. *apetala* and *S. caseolaris* had lower carbon storage in biomass than in the monocultures due to the interspecific competition for light [23]. Similar consequences seem to apply to *K. obovata,* the growth of which was impeded by the relative low light condition under the *S*. *apetala* canopy.

### Organic carbon accumulation in soil

It should be noted that the soil organic carbon detected in our study was lower than previously reported in mangroves. The difference might be due to the original soil substrate, spatial climatic conditions or the age of trees. Soil organic matter comprises the largest C pool in most forests [17]. Soil organic carbon storage represented 58%–87% of the belowground organic carbon storage and contributed the majority of the forest carbon pool in this study. On average, 58% of mangrove soil carbon is derived from litter and root production, which is the major transfer pathway for plant tissue carbon into soil [22, 26, 49, 50]. A significant positive correlation exists between the organic carbon storage in litter and soil in all three mangrove forests. However, SA produced more organic carbon in litter than KO did, while a reversed pattern applies to the soil organic carbon stocks. The organic carbon in roots of the three mangrove forests varied not only with depth but also across mangrove species. Significant positive correlations between organic carbon in roots and soil was also present for all forest types, suggesting that roots may contribute significantly to soil organic carbon accumulation. Roots have a more significant effect than litter on soil composition and vertical soil accretion in mangrove forests [22, 51].

Effects of mangrove species on soil organic matter accumulation depends primarily on their strategies of root production [50]. The soil bulk density in the mangrove forests was lower than that of the adjacent unvegetated mudflat. Compared with KS and SA where *S*. *apetala* was present, the greater roots biomass and soil organic carbon stock of monospecific KO had a lower soil bulk density. The development of mangrove roots and soil organic matter accumulation process may result in a more porous and less compacted substrate. The root system including pneumatophores of *S*. *apetala* may facilitate deposition of organic matter on the tidal flat adjacent to the forest fringe. The lack of pneumatophores may require *K. obovata* to develop more superficial fine roots to enhance gas exchange and nutrient uptake in the anoxic soils. These may facilitate sedimentation and promote soil organic carbon accumulation in the surface soil [23, 52].

The colonisation of *K. obovata* not only diversified the spatial niche utilisation of the forest, but also increased the overall root biomass of the *S*. *apetala* forests. Notwithstanding, colonisation comes with a cost to *K. obovata:* the low light condition resulted in reduced root production of this species in mixed forests compared to the KO monoculture. For belowground carbon accumulation, the rate of root production must exceed that of carbon loss [53]. Root decomposition rate is also a key factor to soil organic carbon accumulation, which is primarily driven by soil characteristic, rainfall, tidal regime and mangrove forest types [47, 54–56]. The large amount of undecomposed root necromass from *K. obovata* reduces the overall root decomposition rate of the mixed forest and may contribute to the higher organic matter accumulation than in the *S*. *apetala* monoculture. Increased root productivity coupled with reduced decomposition in anoxic soils is a dominant driver in carbon sequestration by mangrove forests [20].

### The potential for maximising carbon storage through multi-specific mangrove plantations

Large-scale monospecific plantations of selected species based on growth rate (e.g. *Sonneratia* spp.) or ease of planting (e.g. *Rhizophora* spp.) have dominated the global strategy for mangrove restoration [29]. However, natural colonisation of non-planted species into mangrove monocultures can increase their productivity substantially [57]. Coexistence might also change the carbon allocation strategy of afforested species compared to monospecific plantations [58]. Although our study demonstrates that multi-canopy forests may not necessarily have higher carbon storage than monospecific stands because of interspecific competition, there is potential for managing the interactions to achieve optimal outcomes. Lowering the density of *S*. *apetala* in the mixed forest may alleviate adverse competition effects on *K*. *obovata,* achievable through a proper density matching process during the early stage of afforestation [59, 60]. There is much room for developing the necessary knowledge as well as its application of multi-species mangrove restoration as a central strategy in mangrove restoration.

In China, more than 80% of the mangrove plantations after 2000 are dominated by *S*. *apetala* [33]. In southern China, *S*. *apetala* has naturalised at over 40% of the original natural mangrove forests [46]. As demonstrated by our study, the large extent of *S*. *apetala* monocultures presents great opportunities to improve their ecological services such as carbon storage potential by transforming them into multi-specific forests through introduction of native species with complementary niches.

## Conclusion

Contrary to the hypothese inferred from aboveground morphology and individual appearances of mangrove species, the overall root biomass and organic carbon storage in the *K. obovata* monoculture were significantly higher than those of both the *S*. *apetala* monoculture and mixed forest, suggesting that *K. obovata* should be preferred to *S*. *apetala* for mangrove afforestation programs in the future. Improved spatial niche utilization through the colonization by *K. obovata* in *S. apetala* monocultures promoted organic carbon storage both in biomass and soil. The increase in stem density was a principle factor that the multi-canopy forest supported higher productivity and carbon storage than the monospecific pure stands. Root production and decomposition contributed more than litter dynamics on soil organic carbon accumulation, driving species-specific as well as overall rates of soil organic matter accumulation. For the existing mono-specific plantations, especially for *S*. *apetala* forests, introducing native mangroves may enhance their carbon storage capacity for a long-term perspective.

## Data Availability

The data supporting this project are clearly referenced or available in this paper itself. The data used to produce the figures in this paper can be provided as request.

## Abbreviations

AGB: Above ground biomass
AGOC: Above ground organic carbon
ANOVA: Analysis of variance
BA: Basal area
BGB: Below ground biomass
BGOC: Below ground organic carbon
DBH: Diameter of breast height
GL: Ground layer
GLOC: Ground layer organic carbon
NA: Not applicable
NS: Not significant
SBD: Soil bulk density
SE: Standard error
SOC: Soil organic carbon
SPSS: Statistical Product and Service Solutions
TOC: Total organic carbon
